# Magnetically levitated mesenchymal stem cell spheroids cultured with a collagen gel maintain phenotype and quiescence

**DOI:** 10.1177/2041731417704428

**Published:** 2017-04-24

**Authors:** Natasha S Lewis, Emily EL Lewis, Margaret Mullin, Helen Wheadon, Matthew J Dalby, Catherine C Berry

**Affiliations:** 1 Centre for Cell Engineering, Institute of Molecular, Cell and Systems Biology (IMCSB), The University of Glasgow, Glasgow, UK; 2 Electron Microscopy Unit, The University of Glasgow, Glasgow, UK; 3 Paul O’Gorman Leukaemia Research Centre, The University of Glasgow, Glasgow, UK

**Keywords:** Nanoparticles, mesenchymal stem cells, spheroids, bone marrow, niche

## Abstract

Multicellular spheroids are an established system for three-dimensional cell culture. Spheroids are typically generated using hanging drop or non-adherent culture; however, an emerging technique is to use magnetic levitation. Herein, mesenchymal stem cell spheroids were generated using magnetic nanoparticles and subsequently cultured within a type I collagen gel, with a view towards developing a bone marrow niche environment. Cells were loaded with magnetic nanoparticles, and suspended beneath an external magnet, inducing self-assembly of multicellular spheroids. Cells in spheroids were viable and compared to corresponding monolayer controls, maintained stem cell phenotype and were quiescent. Interestingly, core spheroid necrosis was not observed, even with increasing spheroid size, in contrast to other commonly used spheroid systems. This mesenchymal stem cell spheroid culture presents a potential platform for modelling in vitro bone marrow stem cell niches, elucidating interactions between cells, as well as a useful model for drug delivery studies.

## Introduction

Human mesenchymal stem cells (MSCs) are increasingly important in tissue engineering and regenerative medicine. Their capacity to differentiate into several cell types, including osteocytes, chondrocytes and adipocytes,^1^ promotes their use for orthopaedics applications in particular. MSCs are typically isolated from bone marrow (BM) and are traditionally expanded in culture on polystyrene in a two-dimensional (2D) monolayer. However, one of the major limitations in utilising MSCs ex vivo is that they lose their differentiation potential or undergo replicative senescence after a certain number of population doublings.^2,3^ Additionally, fundamental stem cell properties such as quiescence and multipotency are gradually lost in culture, limiting their usefulness.^4^ There is a growing need to develop MSC culture systems to circumvent these issues.

There is a growing wealth of evidence confirming that the microenvironment MSCs reside within is crucial for maintaining stem cell function and preventing differentiation.^5^ MSCs reside in a protective location termed the ‘niche’. MSC niches are continuously being discovered, and they can be extracted from adipose tissue,^6^ dental pulp^7^ and amniotic fluid,^8^ although these cells have different differentiation capacities and characteristics. The traditional niche for MSC extraction, however, is in the BM. This is a complex environment, and is also home to osteoblasts, endothelial cells, haematopoietic stem cells (HSCs) and macrophages, among others, with cellular interactions contributing to MSC phenotype. The niche also presents extracellular matrix (ECM) and facilitates cell–cell interactions which are not easily replicated ex vivo. The niche architecture acts synergistically to protect MSCs from overstimulation, prevent differentiation and maintain quiescence.^9^ Therefore, the BM niche environment is fundamental to MSC phenotype. Standard tissue culture methods impose an artificial conformation on cells, hence the observed phenotypic changes. More recently developed culture systems aim to provide a three-dimensional (3D) environment with ECM and/or enhanced intercellular interactions in an attempt to mimic the in vivo conditions. In addition to spheroid systems, which produce aggregates of cells in a spherical shape, these include organoids, which comprise several cell types, tissue explant cultures and multiplexed ‘organ on a chip’ models.^10^

Multicellular spheroids are one such 3D culture system which has been used to generate embryonic bodies and artificial tumours for cancer studies for decades.^11,12^ Spheroids are aggregates of cells, which can self-organise and behave more akin to cells in vivo, replicating tissue organisation and developing concentration gradients of nutrients and cytokines. For example, as spheroids closely mimic tumour architecture, mirroring pathophysiologically relevant cytokine and nutrient gradients, generating 3D ECM features and promoting secondary central necrosis,^13^ they allow a more reliable test platform for anti-tumour drugs. Spheroids can be formed via several techniques, including spinner flasks,^12^ hanging drops^14^ and culture in low-adherence vessels.^15^ However, these methods are often costly and time consuming. Magnetic cell levitation^16^ is a recent method which has been developed, which offers an alternative method for spheroid generation. During magnetic levitation, cells are pre-loaded with magnetic nanoparticles and encouraged to form multicellular spheroids within several hours via an external magnetic field. The resultant spheroids are easily manipulated by virtue of their magnetic qualities and also confer benefits including cell tracking and imaging.

The ECM is an important part of the MSC niche.^17^ This network of proteins is comprised of collagen type I, III and IV; fibronectin; laminin; thrombospondin; hemonectin; and proteoglycans.^18,19^ In particular, collagen type I localises to the endosteal surface and endosteal marrow, the proposed stem cell niche site, and is the most abundant type of collagen in the marrow.^19^ These molecules present topographical and biological signals to the cells and allow for enhanced cell adhesion.^9^ The incorporation of these proteins into a 3D culture system may further encourage maintenance of MSC phenotype. In addition, the 3D fibrous network would better mimic small molecule diffusion through the BM, an important factor to consider when assessing drug delivery mechanisms.

Here, we have developed a spheroid culture system in which MSCs are labelled with magnetic nanoparticles, multicellular spheroids are then generated and subsequently implanted into a type I collagen gel with a similar stiffness (modulus of 36 Pa)^20^ to the in vivo BM microenvironment (modulus of 100 Pa).^21,22^ The resultant magnetic MSC niche cultures can be maintained within a multiwell plate format. In this study, we have characterised spheroid formation in terms of nanoparticle uptake and initial cell seeding density. We have shown that spheroid formation is reproducible, generating spheroids that are distinct, viable and do not suffer from excessive central necrosis. We further showed that spheroid cultured within a collagen gel encourages a quiescent, non-proliferative cell state, as in vivo. This MSC BM niche model presents an attractive tool for MSC studies in vitro and a useful platform for pharmaceutical testing.

## Materials and methods

### Cell culture

Human mesenchymal stem cells from bone marrow (hMSC-BM) were purchased from Promocell GmbH (Heidelberg, Germany) and cultured in high-glucose Dulbecco’s Modified Eagle’s Medium (DMEM, Sigma-Aldrich, St Louis, MO, USA) supplemented with 10% foetal bovine serum (FBS), 2% antibiotic mixture (penicillin–streptavidin, glutamine, fungizine), 1% non-essential amino acids and 1% sodium pyruvate (Sigma-Aldrich) at 37°C, 5% CO_2_. MSCs were used for experiments between passages 1 and 4.

#### Magnetic cell levitation and spheroid formation

MSCs were seeded into 24-well plates at densities of 1 × 10^4^, 5 × 10^4^ or 1 × 10^5^ cells mL^−1^, equivalent to 5200, 26,000 and 52,000 cells cm^−2^, respectively. After 24 h, green fluorescently labelled (excitation wavelength 502 nm, emission wavelength 525 nm) magnetic iron oxide (FeO_3_) 200 nm diameter nanoparticles (Chemicell GmbH, Berlin, Germany) were added to the adhered cells at a concentration of 0.1 mg mL^−1^ and subsequently incubated for 30 min directly above a magnetic plate to encourage cell internalisation. Cells were then washed with HEPES saline three times to remove excess nanoparticles and detached via trypsin. The resulting cell suspension was centrifuged (4 min, 445*g*), the supernatant was removed and the cells were re-suspended in fresh medium. This suspension was then transferred to 6-well plates at a concentration of 1 × 10^4^ cells mL^−1^, with a single neodymium magnet (15 × 5 mm, 3500 Gauss/350 mT; First4magnets, UK) fixed to the culture plate lid above each well to attract the magnetically labelled cells together; the magnets were at a distance of 15 mm above the base of the well, and 5 mm above the surface of the culture medium. Spheroids formed within 24 h.

#### Spheroid transfer into collagen type I gel

After 24 h, the spheroids were transferred into another 24-well plate prior to the addition of 1 mL collagen gel solution (approximately 8.3% 10× DMEM (FirstLink, Wolverhampton, UK), 8.3% FBS, 8.3% DMEM, 41.6% collagen (2.05 mg mL^−1^, FirstLink), adjusted to pH 8.2 with 0.1 M NaOH). The plate was placed in an incubator to allow gelation to occur at 37°C. A magnet was placed over each well for the first 24 h of culture.

### Scanning and transmission electron microscopy

Spheroid samples were prepared for scanning electron microscopy (SEM) to observe gross spheroid morphology and transmission electron microscopy (TEM) to allow ultra-structural observations after 3 days of culture. In brief, samples were fixed in 2.5% glutaraldehyde in 0.1 M sodium cacodylate (SC) buffer for 1 h. Cells were washed three times in 0.1 M SC and incubated for 1 h in 1% osmium tetroxide. Samples were rinsed three times in distilled water before 1 h incubation in 0.5% aqueous uranyl acetate in the dark. Spheroids were washed a further two times in distilled water and then subjected to dehydration through an alcohol series before being critical point dried. SEM samples were then mounted onto stubs, sputter coated in gold and imaged on a scanning electron microscope, while TEM samples were embedded in resin and sectioned prior to imaging on a transmission electron microscope.

### Cell viability staining

Cell viability was determined using an ethidium homodimer/calcein AM viability kit (Life Technologies, Carlsbad, CA, USA) which stains live cells green and dead cells red. After 48 h of spheroid implantation into collagen gel, culture media containing 1 µL mL^−1^ of each of stain was added to live cells and cultures were returned to the incubator. After 1 h, the cells were washed with warmed fresh media and immediately imaged using a fluorescence microscope.

#### Image analysis

For viability images, a threshold was set for the fluorescent signal in FIJI for both fluorescein isothiocyanate (FITC) and tetramethylrhodamine (TRITC) channels so that discrete cells were distinguishable. A watershed was set to discriminate between individual cells in clumps, and cell number and total amount of fluorescence was measured using the ‘particle analysis’ function. The cell numbers and number of fluorescing pixels from TRITC and FITC filters were combined for each spheroid.

Cross-sectional signals for FITC and TRITC were obtained using the ‘plot profile’ function. Intensity values obtained from each channel were plotted against distance for each spheroid. Spheroid size was evaluated by drawing an area around each spheroid and using the ‘measure’ function in FIJI.

### Immunohistochemistry

Cultures were initiated, and MSC spheroids were grown for 7 days before being fixed for 15 min at 37°C with 4% formaldehyde, 2% sucrose in phosphate-buffered saline (PBS). They were incubated in permeabilising buffer (10.3 g sucrose, 0.292 g NaCl, 0.06 g MgCl_2_ (hexahydrate), 0.476 g HEPES, 0.5 mL Triton X in 100 mL PBS, pH 7.2) for 5 min at 4°C. Samples were blocked in 1% bovine serum albumin (BSA)/PBS and stained with primary antibody (Table 1) diluted in 1% BSA/PBS for 1 h at 37°C. After the incubation, they were washed three times in PBS/0.5% Tween 20. They were then incubated with a biotin-conjugated secondary antibody (1:200 in 1% BSA/PBS, Vector Laboratories, mouse or rabbit) for 1 h at 37°C. After three washes in PBS/0.5% Tween 20, streptavidin-FITC (1:50 in 1% BSA/PBS, Vector Laboratories) was added and incubated for 30 min at 37°C. The samples were then washed three more times in PBS/0.5% Tween 20 and mounted in DAPI. An Axiovert 200 microscope was used to image the samples.

**Table 1.** Primary antibodies used for immunohistochemistry.

Primary antibody	Manufacturer	Dilution	Secondary antibody
Anti-nestin	Santa Cruz Biotechnologies, Dallas, TX, USA	1:100	Mouse
Anti-STRO-1	Santa Cruz Biotechnologies	1:50	Mouse
Anti-SDF-1α	Abcam, Cambridge, UK	1:200	Rabbit

Rhodamine phalloidin (Life Technologies, ThermoFisher Scientific, Waltham, MA, USA) was used to stain actin filaments on fixed samples for fluorescence microscopy if appropriate. At the primary antibody incubation stage, phalloidin diluted 1:500 in 1% PBS/BSA was added.

### Gene expression analysis

RNA isolation and gene expression analyses were performed as previously described.^20^

#### RNA isolation

Analysis was conducted on MSCs cultured both as monolayer and spheroid culture systems for either 1 or 14 days. For the monolayer cultures, three samples were pooled for each replicate (n = 3), whereas for the spheroid cultures, six samples were pooled for each replicate (n = 3).

In brief, cells were lysed with 1 mL TRIzol (ThermoFisher Scientific) for 10 min at room temperature and centrifuged at 4°C (12,000*g* for 15 min). The supernatant was mixed thoroughly with 200 µL chloroform and incubated at room temperature (3 min). The mixture was centrifuged with the same parameters, the aqueous phase was removed, and then glycoblue (1 µL) and isopropanol (500 µL) were added to the solution. The tubes were inverted several times, and then incubated at room temperature (10 min), followed by centrifugation at 4°C (12,000*g* for 20 min). The supernatant was removed, leaving a blue pellet, which was vortexed with 1 mL ethanol (25% aq) and then centrifuged at 4°C (7500*g* for 5 min). The ethanol was removed to air dry the pellet and then RNase-free water (20 µL) was added and the samples were incubated at 55°C (10 min). The samples were then further processed using a Qiagen (Manchester, UK) RNeasy micro kit according to the manufacturer’s instructions.

#### Fluidigm preparation

RNA samples were subjected to reverse transcription using SuperScript III Reverse Transcriptase (ThermoFisher Scientific) as previously described. At all stages of the process, reactions were performed at 4°C unless stated. In all, 11 µL of each sample was added to 1 µL of oligo (dT) and 1 µL dNTPmix and then heated to 65°C for 5 min. A mixture containing 4 µL 5× First Strand buffer, 1 µL 0.1 M DTT, 1 µL RNaseOUT Recombinant RNase inhibitor, 0.5 µL SuperScript III RT and 0.5 µL water was prepared and added to each sample and left for 5 min. The solution was then incubated at 50°C for 30 min followed by 70°C for 15 min to produce cDNA. All 48 primers (see Supplementary Table 1) were pooled together (1 µL from each primer set with 152 µL of DNA suspension buffer). A new solution was prepared with 1.25 µL from the cDNA of each sample, 2.5 µL 2× TaqMan PreAmp Master Mix, 0.5 µL pooled primer mix and 0.75 µL water. These samples were vortexed, centrifuged and subjected to 22 thermal cycles as detailed in Table 2.

**Table 2.** Thermal cycler conditions used on each sample prior to Fluidigm analysis.

Condition	Hold	22 Cycles		Hold
Temperature	95°C	95°C	60°C	4°C
Time	10 min	15 s	4 min	∞

After the 22 thermal cycles, 1.4 µL water, 0.2 µL Exonuclease I Reaction Buffer and 0.4 µL exonuclease were added to each sample and vortexed, centrifuged and incubated at 37°C for 30 min and then at 80°C for 15 min. After heating, 18 µL of TE buffer was added to each sample. The Exonuclease I treated sample (2.7 µL) was added to 3.0 µL 2× SsoFast EvaGreen Supermix (Bio-Rad Laboratories, Hercules, CA, USA) and 0.3 µL 20× DNA Binding Dye sample loading reagent. Each sample was vortexed and centrifuged then loaded onto the chip. Additionally, 0.3 µL of each individual primer set was added to 3 µL 2× assay loading reagent, and 2.7 µL 1× DNA suspension buffer was vortexed and centrifuged prior to loading on the chip. A 48.48 Dynamic array IFC was used during this analysis.

### Rheology

Gels were prepared and analysed after 3 days incubation. Analysis was carried out at 25°C within a heat-controlled environment and with a parallel plate (20 mm diameter). Additionally, a solvent trap was used to minimise solvent evaporation, thus creating a saturated internal atmosphere. A strain sweep of the gels was initially used to ensure elastic modulus (G′) and viscous modulus (G″) measurements were taken within the linear viscoelastic region. Frequency sweeps of the gel were carried out between 0.1 and 40 Hz to determine the dynamic modulus of the gel. All analysis was conducted using a Malvern Kinexus rheometer.

### Histological analysis

MSCs were grown in spheroids, both implanted into a collagen gel and grown in media only. After 7 days, the spheroids in gel were treated with collagenase D (Roche, 90 min, 2 mg mL^−1^, equal volume). All spheroids were then dissociated using resuspension with a needle and re-seeded onto sterilised glass coverslips. The following day, the media was changed to adipogenic, osteogenic or chondrogenic induction media (DMEM with 10% FBS and 2% antibiotics, with supplements as listed in Table 3) and the cells were grown for 30 days, with media changed twice a week. Cells were fixed and stained with Oil Red O, Alizarin Red or Safranin O stains, respectively. MSCs grown in monolayers for 30 days were used as a control.

**Table 3.** Supplements used for differentiation media formulations.

Induction media	Supplements
Osteogenic	350 µM ascorbate-2-phosphate, 0.1 µM dexamethasone
Adipogenic	1 µM dexamethasone, 1.7 nM insulin, 200 µM indomethacin, 500 µM isobutylmethylxanthine
Chondrogenic	10 ng mL^−1^ TGFβ1, 100 nM dexamethasone, 6.25 µg mL^−1^ insulin, 50 nM ascorbate-2-phosphate, 100 mg L^−1^ sodium pyruvate

### Oil red O staining

Samples were rinsed in 60% isopropanol and stained in Oil Red O for 15 min. They were then rinsed in isopropanol until colourless, followed by a wash with water. The nuclei were counterstained with Weigert’s haematoxylin for 10 min, washed again with water and mounted onto slides with an aqueous mountant.

### Alizarin red staining

The samples were washed in graded alcohols to water and then stained with Alizarin Red solution for 5 min. The samples were blotted with filter paper and then rinsed in acetone for 30 s. They were then rinsed in a 1:1 solution of acetone-xylene for 15 s, followed by a wash with xylene only. They were then mounted onto slides with aqueous mountant.

### Safranin O staining

Samples were taken to water through three changes of graded alcohols. They were then stained with Weigert’s haematoxylin for 10 min, followed by a wash in water for 10 min. They were then stained with 0.001% Fast Green for 5 min, rinsed in 1% acetic acid for 10 s and stained with 0.1% Safranin O solution for 7 min. This was followed by a wash with water. The samples were dipped in 1% acetic acid and then dehydrated and mounted onto slides with aqueous mountant.

### Statistical analyses

Analysis of variance (ANOVA) was performed in GraphPad Prism. Correlation coefficients were calculated using the CORREL function in Microsoft Excel. Graphs were plotted in either GraphPad Prism or Microsoft Excel.

## Results and discussion

### Confirmation of nanoparticle internalisation and spheroid formation using electron microscopy

**Figure 1.**
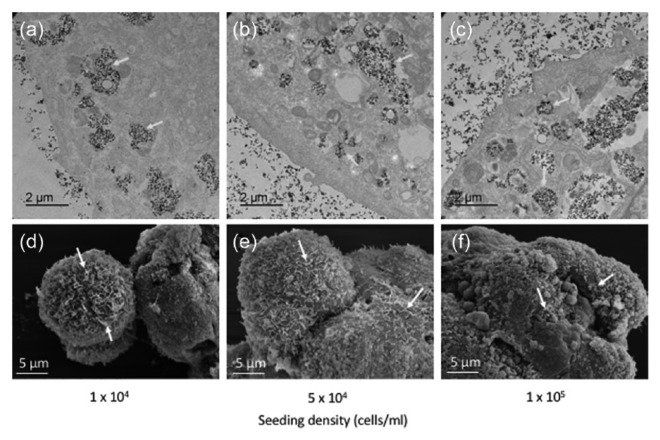
Multicellular MSC spheroids generated using different cell seeding densities. (a–c) Transmission electron micrographs clearly indicate nanoparticles located in vesicle within the cell cytoplasm, while (d–f) scanning electron micrographs show the general morphology and dimensions of the multicellular MSC spheroids. Yellow arrows, nanoparticles within vesicles; white arrows, cellular projections.

TEM and SEM (Figure 1) were used to evaluate nanoparticle uptake, spheroid formation and morphology, and spheroid ultrastructure at different cell seeding densities. The TEM images clearly showed nanoparticle internalisation into the MSCs, which were located in vesicles within the cytoplasm (Figure 1(a)–(c), yellow arrows). There was no apparent difference in cellular uptake of nanoparticles between the different seeding densities.

The SEM images indicate that the spheroids, once formed, develop into spherical structures made up of multiple cellular domains (Figure 1(d)–(f)). This general spheroid shape did not differ between the different seeding densities, although larger aggregates of several spheroid structures were observed at the higher densities. These images confirm that the spheroid formation remains constant regardless of cell number. Multiple cellular projections were visible on the cell surface (indicated by white arrows), reflecting cellular interaction with the 3D environment.

### MSC spheroid size increases relative to cell number

**Figure 2.**
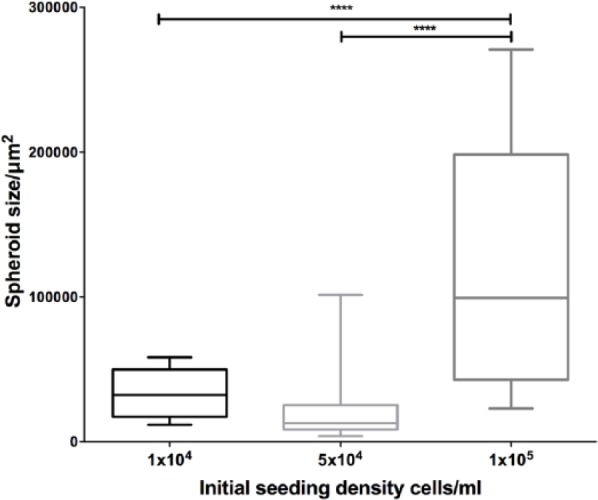
Spheroid size variation using different seeding densities. Spheroid cross-sectional area in µm^2^ was measured for all spheroids imaged at each time point: 1 × 10^4^, n = 11; 5 × 10^4^, n = 37; 1 × 10^5^, n = 17. Statistical significance is based on results of one-way ANOVA. ****p < 0.0001.

MSC spheroids generated using different seeding densities were measured in terms of their cross-sectional area (CRA) in FIJI (Figure 2). The differences in spheroid size at 1 × 10^4^ and 5 × 10^4^ cells mL^−1^ are not statistically significant; however, a large significant increase in size was observed for spheroids seeded at 1 × 10^5^ cells mL^−1^. The range of sizes at this higher density was varied, with many smaller spheroids apparent, similar to those at the lower seeding densities. When considered with the SEM images in Figure 1, this suggests that there may be a limit on spheroid size when formed via magnetic levitation, and the larger structures that were observed result from amalgamation of several smaller ones.

Compared to other MSC spheroid systems generated using different methods, these spheroids are smaller than expected, given that the seeding density we used. For example, Bartosh et al.^23^ reported MSC spheroids, generated through the hanging drop method, of approximately 400 µm diameter, equivalent to a CRA of approximately 0.13 mm^2^, when cells were seeded at a density of 10,000 cells per drop. This is compared to a median CRA of around 0.025 mm^2^ for our spheroids when seeded with the same number of cells. Similarly, cells seeded at 100,000 cells per hanging drop had an approximate CRA of around 0.64 mm^2^ (diameter of around 900 µm) compared to 0.2 mm^2^ for our magnetically levitated spheroids.

Rossi et al.^24^ used a non-adherent surface to generate MSC spheroids. Similarly, the spheroids from this system were larger than those created through magnetic levitation (50,000 cells produced spheroids of 0.26 mm^2^, compared with 0.025 mm^2^ in our study), although they were more comparable to those that were magnetically levitated. This 10-fold difference may be partly due to cell loss during the levitation process, in addition to the formation of several different spheroids.

Zimmermann et al.^25^ produced spheroids that were slightly smaller but comparable in size to magnetically levitated spheroids, using forced aggregation followed by centrifugation. Given that these were initiated with an order of magnitude fewer cells than with our method, this indicates that our method produces smaller spheroids than expected with other methods.

A possible explanation for these size differences is that in a hanging drop system especially, the cells are forced to aggregate into a single structure. In the magnetic levitation method, cells are forced together, but this results in a collection of smaller aggregates each of under 1000 cells individually (Figure 2). These smaller spheroids may be more similar in structure to collections of cells within the BM compared with larger spheroids, as the diameter of BM sinusoids around which MSCs are found is up to 60 µm,^26^ equivalent to a CRA of 2800 µm^2^. Although the smallest spheroids produced are larger than BM sinusoids, their conformation may be more similar to MSC clusters in vivo than the conformation of the bigger spheroids.

**Figure 3.**
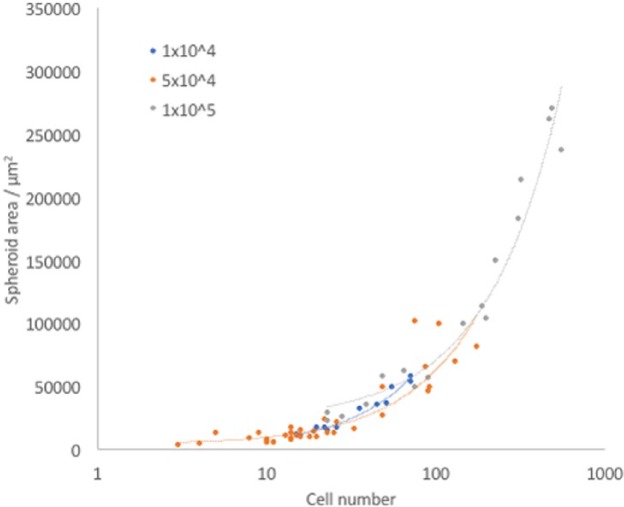
Spheroid size increases with increasing cell number. Spheroids are plotted by cell number against size. The Pearson Product–Moment Correlation Coefficient between spheroid area and cell number was calculated for each seeding density using the CORREL function in Excel: 1 × 10^4^, 0.98206805; 5 × 10^4^, 0.880558; 1 × 10^5^, 0.9755847.

Strong positive correlation was observed between spheroid size and spheroid number at all densities, in accordance with expectations (Figure 3). The largest spheroids were made up of over 500 cells individually. It is worth noting that the larger spheroids were less spherical and more cylindrical in shape compared to the smaller ones, due to the non-uniform properties of the magnetic field, as characterised previously in our lab.^27,28^ A cylindrical shape may contribute to preventing central necrosis by decreasing the distance in one plane from the cells in the centre to the edge of the spheroid. This means that diffusion of factors from the media to the cells is enhanced and the cells are less likely to die from starvation. The same is true of oxygen: although hypoxia would not necessarily kill the cells directly, the concentration of oxygen in the centre of a cylindrical spheroid will be more similar to that experienced by cells at the edge, compared to cells in the centre of a spherical spheroid with the same cell number.

### MSC spheroid viability is maintained with increasing cell density

**Figure 4.**
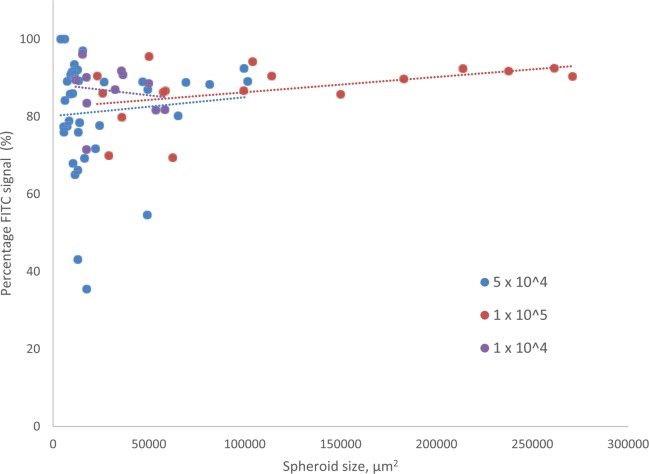
Percentage viability of spheroids in relation to spheroid size. Percentage FITC signal detected for each spheroid is plotted against spheroid cross-sectional area in µm^2^. The Pearson Product–Moment Correlation Coefficient between spheroid size and percentage viability was calculated for each seeding density using the CORREL function in Excel: 1 × 10^4^, −0.145204; 5 × 10^4^, 0.0885486; 1 × 10^5^, 0.4547947.

The percentage of pixels producing signal, in a single plane cross through the centre of a spheroid, section above a given threshold in the FITC channel as compared to those producing signal in the TRITC channel was calculated as a proxy for percentage viability for each spheroid. There was no statistically significant difference in percentage viability in spheroids seeded at the different densities (Supplementary Figure 1). The relationship between percentage viability and spheroid size is shown in Figure 4. Correlation between the two is negligible for the lowest two densities, and positive for the highest density, showing that viability remains high regardless of spheroid size.

### MSC spheroids do not suffer from excessive central necrosis

**Figure 5.**
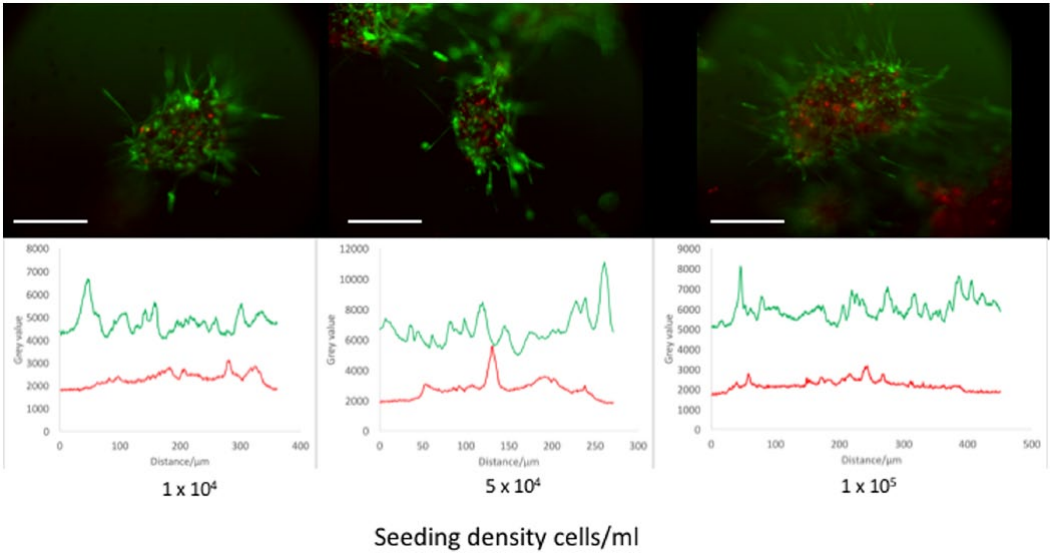
The cross-sectional viable (green; FITC) and dead (red; TRITC) fluorescent signal in spheroids with different cell seeding densities. Peaks in the graphs indicate an increase in signal. Note the higher level of FITC signal throughout the spheroid compared to TRITC, which has occasional peaks, more towards the centre of the spheroid. Scale bar = 200 µm.

Many spheroid systems suffer from central necrosis when they reach a certain size threshold, due to increased diffusion distance for vital nutrients and potential internal hypoxia.^13,14,16^ To assess the extent of necrosis in this magnetic MSC spheroid system, cross-sectional signals of TRITC and FITC were taken to identify clusters of dead and live cells, respectively. Figure 5 displays these cross-sections alongside fluorescence microscope images of spheroids formed from cells seeded at different initial seeding densities. For all densities, TRITC signal is maintained at a low level with no elongated peaks, which would indicate clusters of dead cells (central necrosis). On the other hand, FITC signal is maintained at a high level, indicating constant high viability across the rest of the spheroid.

The diameter of the spheroids generated from our magnetic levitation method range between 200 and 360 µm (median size for the lowest and highest seeding densities, respectively). We did not detect areas of central necrosis in these spheroids. It has been reported that spheroids of diameters greater than 400–600 µm will exhibit central necrosis, at twice the thickness of the viable cell rim.^29^ As our spheroids are below this threshold, they conform to this model and do not exhibit excessive central necrosis.

### MSCs in spheroids maintain expression of stem cell markers

**Figure 6.**
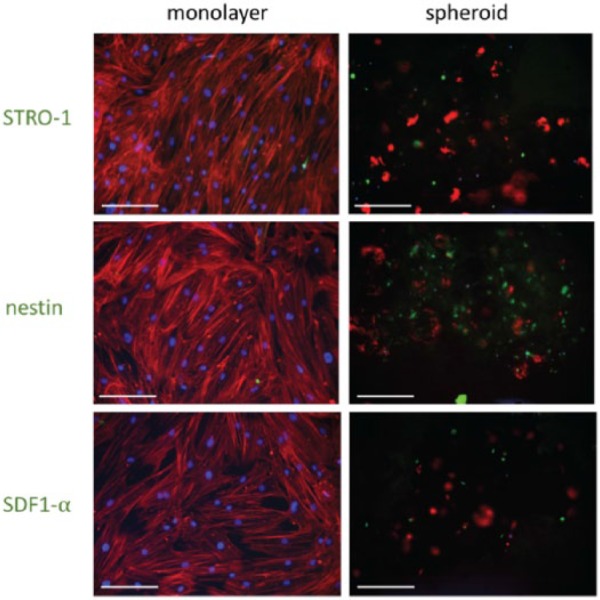
Immunohistochemistry performed on MSCs grown in either monolayer or spheroid culture systems for 7 days. DAPI (blue) = DNA/nuclei, TRITC (red) = phalloidin (actin); FITC (green) = STRO-1, nestin or SDF1-α, respectively. Scale bars = 200 µm.

MSCs grown in either monolayers or spheroids in media for 7 days were analysed using immunohistochemistry to investigate retention of stem cell properties (Figure 6).

STRO-1 is a marker used to isolate MSC colony forming units (CFU-C).^30^ However, MSCs in standard 2D culture gradually lose STRO-1 expression.^31^ The results presented here show that MSCs in spheroid cultures retain STRO-1 expression for up to 7 days in culture, whereas it is lost in corresponding MSC monolayer cultures. 2D cultures encourage differentiation, with accompanying loss of these markers. Retention of STRO-1 suggests that the spheroid model system prevents differentiation and maintains the stem cell population as it would be in the BM.

Nestin staining was increased in spheroids compared to monolayers. Nestin is a cytoskeletal component that potentially defines the HSC-supportive subset of MSCs in the BM.^32^ The importance of nestin in the BM niche is now much clearer,^32,33^ and its expression by the MSCs in spheroids is promising, as it indicates that the functionality of the model may be similar to the in vivo niche. These results also suggest that MSCs require 3D culture and/or intimate cell–cell interactions to express nestin and hence support HSCs. SDF1-α is a homing factor that is recognised by the CXCR4 receptor on the HSC surface. It is involved in HSC mobilisation and homing to the BM niche.^34^ The higher expression observed in spheroids compared to monolayers indicates that HSCs are more likely to home to the MSCs cultured as spheroids and that the culture system successfully influenced the expression of this key cytokine.

### MSC cultured as spheroids exit the cell cycle and become quiescent

**Figure 7.**
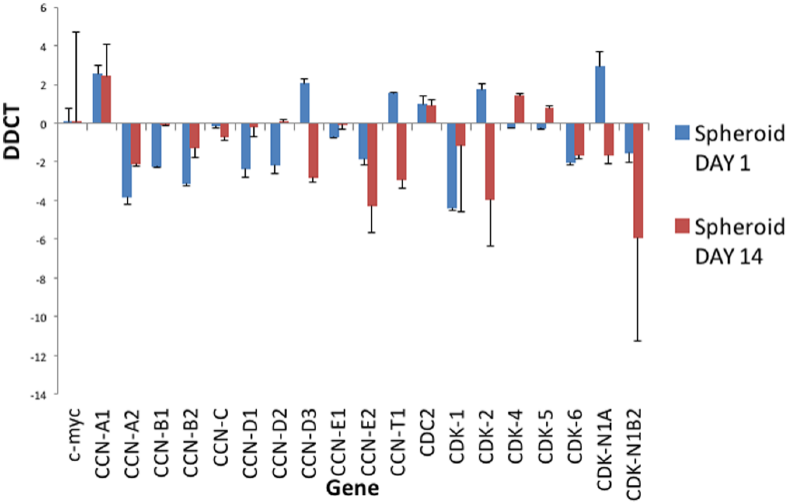
Analysis of expression of cell cycle genes in spheroids. Cell cycle gene analysis of spheroids at day 1 and day 14 normalised to corresponding monolayers. This graph shows expression of cyclins and cyclin-dependent kinases involved within the cell cycle process. This graph shows the fold change in gene expression, identified by Delta-Delta-Ct (DDCT) analysis, in spheroids compared to monolayer culture. CCN = cyclin and CDK = cyclin-dependent kinase. n = 3, technical replicates.

Progression through the cell cycle was evaluated using analysis of gene expression, focusing on key cyclins (CCNs) and cyclin-dependent kinases (CDKs), which have important roles in regulation of the cell cycle. RNA was extracted from MSCs grown in either monolayers or spheroids at day 1 and day 14. The results in Figure 7 show the fold change in gene expression of selected genes involved in the cell cycle in MSC spheroid culture normalised to monolayer culture. Most of these genes are downregulated at day 1, with a further decrease at day 14, indicating that MSCs in spheroids are not progressing as rapidly through the cell cycle.

Cyclins and CDKs form complexes that regulate the transitions between phases of the cell cycle. Cyclin C and CDK3 are involved in the transition between non-proliferative G0 and G1, enabling cells to re-enter the cell cycle.^35^ The expression of cyclin C is slightly decreased in spheroids compared to monolayers at both time points, indicating that more cells are being retained as quiescent in G0.

D-type cyclins (D1, D2 and D3) all mediate the transition from G1 to S phase, during which DNA replication is initiated in preparation for cell division, by forming complexes with CDK4^36^ and CDK6.^37^ Accumulation of cyclin Ds is essential for passing the restriction point in G1 (R), which facilitates S phase, and hence the further phases of the cell cycle, to proceed without receiving further initiating signals. These cyclin D complexes have the capacity to phosphorylate retinoblastoma (Rb), the unphosphorylated form of which is an inhibitor of G1 progression. At day 1, levels of D1 and D2 cyclins in spheroids were significantly lower than in monolayers, although they returned to similar levels at day 14. Cyclin D levels were increased in spheroids at day 1, but decreased significantly at day 14. CDK4 levels are slightly decreased at day 1, but slightly increased at day 14. CDK6 levels are decreased at both time points. These data suggest that insufficient cyclin D accumulation occurs for the cells in spheroids to pass the R point and hence enter S phase.

Similarly, cyclin E complexes with CDK2 and has a role in G1/S transition. It also participates in Rb phosphorylation, and activity is highest during late G1 and early S phase: expression of cyclin E and CDK2 indicates that a cell is committed to cell division. Cyclin E2 is downregulated in spheroids at both time points. CDK2 is slightly increased at day 1 of culture, but is decreased at day 14, indicating that fewer cells are entering S phase.

## Conclusion

Here, we have characterised a 3D MSC culture system comprising of multicellular spheroids, generated via magnetic levitation, cultured within a type I collagen gel. We have observed nanoparticle internalisation and demonstrated that spheroid formation is reproducible, creating spheroids that are distinct, viable and do not exhibit excessive central necrosis. We further showed that the spheroids retain the functionality of BM niche cells, maintaining expression of stem cell markers, releasing haematopoietic maintenance factors and downregulating cell-cycle progression genes. The potential for manipulation due to magnetic properties make these spheroids particularly attractive for 3D systems. This MSC BM niche model provides a simple and relevant platform for pharmaceutical testing and to further allow detailed in vitro studies of MSC within an appropriate 3D environment.

## Supplementary Material

Supplementary material

Supplementary material
