# Supplementary material for: Magnetically levitated mesenchymal stem cell spheroids cultured with a collagen gel maintain phenotype and quiescence
Source: J Tissue Eng. 2017 Apr 24;8:2041731417704428. doi: 10.1177/2041731417704428 (PMC5460809; doi:10.1177/2041731417704428)
Supplement: Supplementary material [file Supplementary_Figures.pdf]

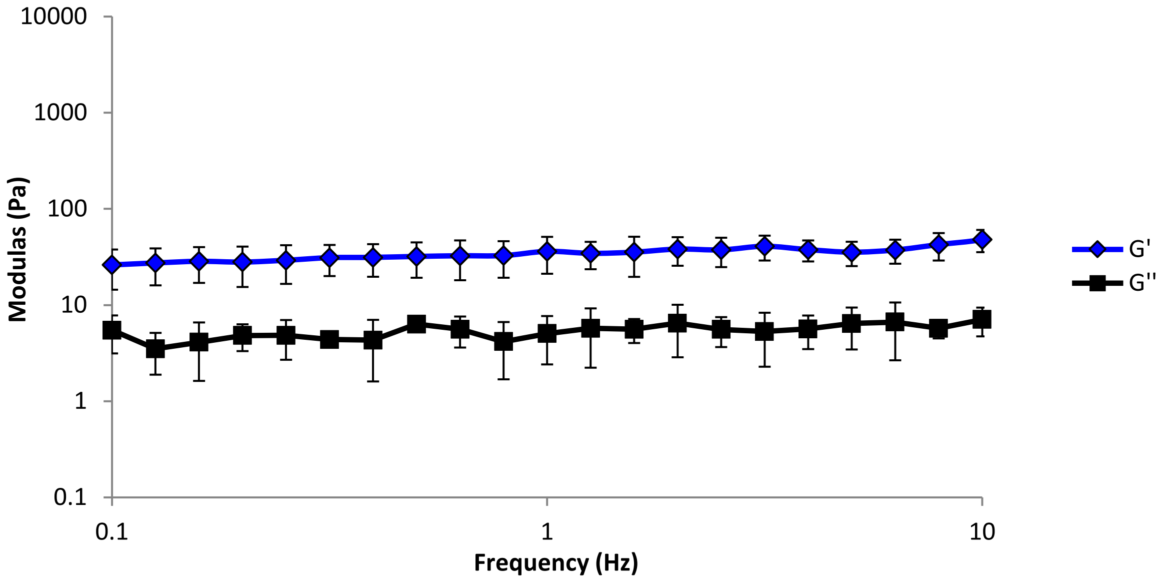

Supplementary Figure 1. Rheological data for collagen type I gels used in this study. G', elastic modulus; G”, viscous modulus.

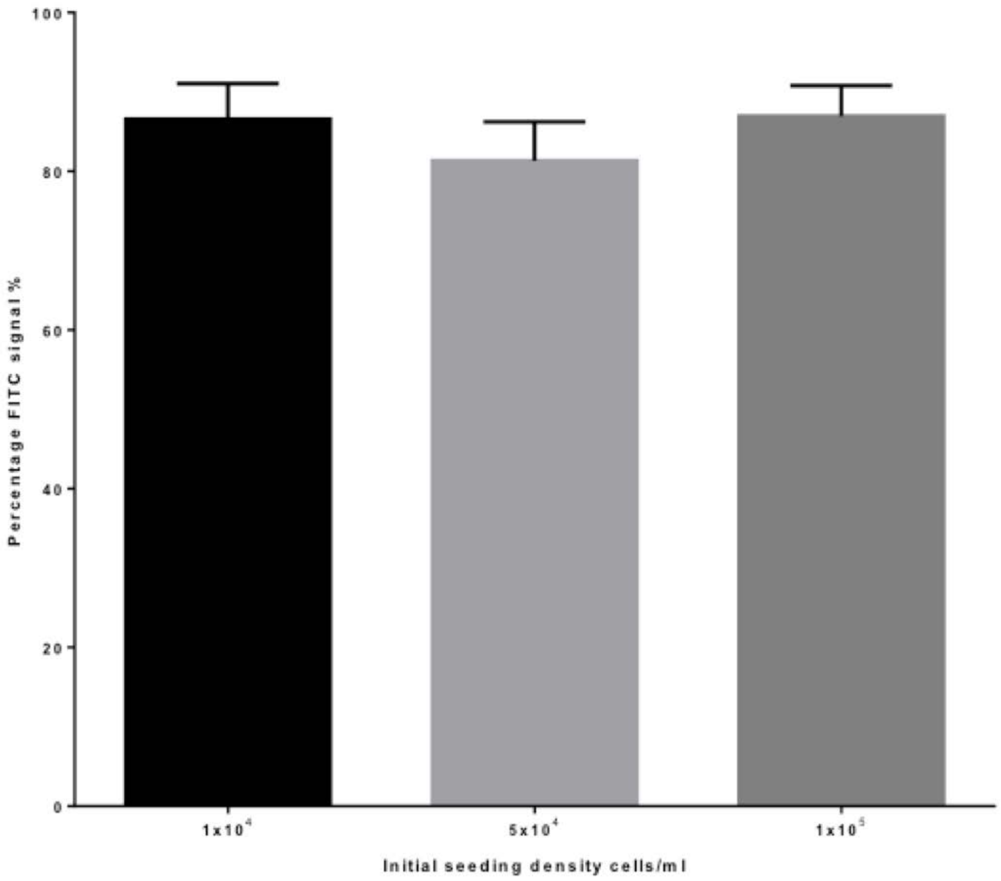

Supplementary Figure 2. Percentage FITC signal from spheroids formed from different initial cell densities following staining for viability (calcein/ethidium homodimer). Green signal (FITC channel) indicated live cells and red signal (TRITC channel) dead cells. Percentage FITC signal was calculated to give a proxy for percentage viability. One-way ANOVA was used to compare the means of all three groups: no statistically significant differences were detected ($p > 0.05$). Percentage viability ± 95% confidence interval.

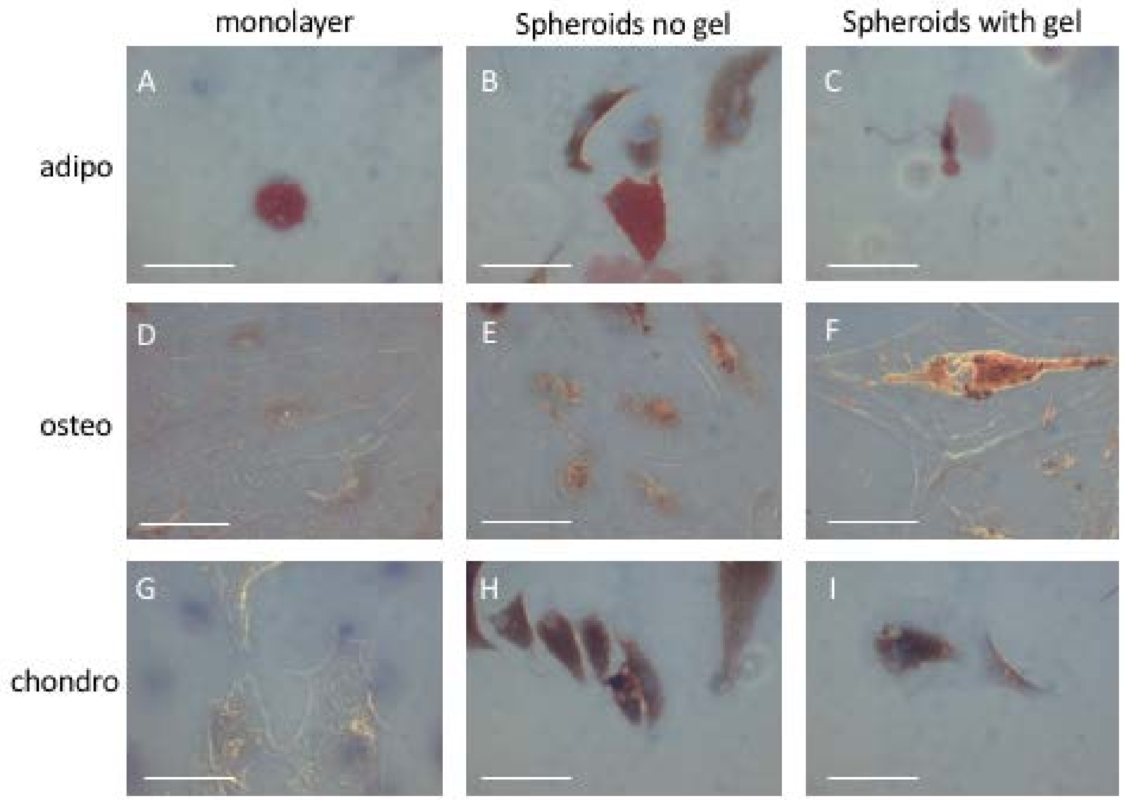

Supplementary Figure 3. Histological analysis of cells grown in adipogenic (A-C), osteogenic (D-F), or chondrogenic (G-I) induction media formulations. A,D,G, MSCs grown in monolayers; B, E, H, MSCs extracted from spheroids grown in media; C, F, I, MSCs extracted from spheroids grown in collagen type I gel. A-C, stained with oil red o; D-F, stained with alizarin red; G-I, stained with safranin O. Scale bar = 100 µm.

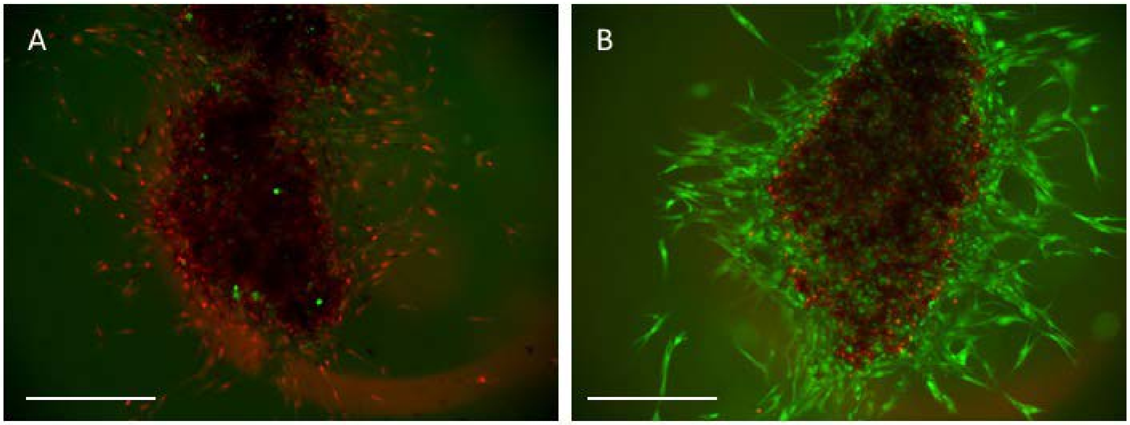

Supplementary Figure 4. Viability staining of (A) a spheroid treated with methanol for 15 min prior to staining with 1 µl/ml calcien/ethidium homodimer; (B) an untreated spheroid exhibiting central necrosis. Viable cells = green; dead cells =red. Scale bar = 400 µm.
