# Supplementary material for: Magnetically levitated mesenchymal stem cell spheroids cultured with a collagen gel maintain phenotype and quiescence
Source: J Tissue Eng. 2017 Apr 24;8:2041731417704428. doi: 10.1177/2041731417704428 (PMC5460809; doi:10.1177/2041731417704428)
Supplement: Supplementary material [file Supplementary_Table_1.pdf]

Supplementary Table 1. Fluidigm primers designed for human genes.

(*Primers used as housekeeping genes).

Primer	Sequence (5’ to 3’)
c-myc	Forward GACTCTGAGGAGGAACAAGA<br>Reverse TTGGCAGCAGGATAGTCCTT
CCNA1	Forward TCAGTACCTTAGGGAAGCTGAAA<br>Reverse CCAGTCCACCAGAATCGTG
CCNA2	Forward CCATACCTCAAGTATTTGCCATC<br>Reverse TCCAGTCTTTCGTATTAATGATTCAG
CCNB1	Forward ACATGGTGCACTTTCCTCCT<br>Reverse AGGTAATGTTGTAGAGTTGGTGTCC
CCNB2	Forward GAAGATTGGGAGAACCCTCA<br>Reverse TGTGGGTTTATGGACTGCAA
CCNC	Forward TTTGCTGAGCTTTCTGTGGA<br>Reverse AATGGTTGCCATCTCTTTTCTC
CCND1	Forward TGCATCTACACCGACAACTCC<br>Reverse CGGATGATCTGTTTGTTCTCCG

CCND2	Forward TACACCGACAACTCCATCAAGC<br><br>Reverse ATGTGCTCAATGAAGTCATGAGG
CCND3	Forward TACACCGACCACGCTGTCT<br><br>Reverse GAAGGCCAGGAAATCATGTG
CCNE1	Forward ACAGCTTGGATTTGCTGGAC<br><br>Reverse TCTGCTTCTTACCGCTCTGTG
CCNE2	Forward GCCATTGATTCATTAGAGTTCCA<br><br>Reverse TGAAATACTGTCCCACTCCAAAC
CCNT1	Forward GAACATGTCATCAAGGTAGCACA<br><br>Reverse AATGACCAGATCTTGAACTTGTTG
CDC2	Forward TGAAATGTTCATGGGGGAAC<br><br>Reverse AAAAAGCTCCTGATGCCTTG
CDK1	Forward AACTACAGGTCAAGTGGTAGCC<br><br>Reverse GGAATCCTGCATAAGCACATCC
CDK2	Forward CCTCCTGGGCTGCAAATA<br><br>Reverse CAGAATCTCCAGGGAATAGGG
CDK4	Forward AGTGTTGGCTGTATCTTTGCAG

	Reverse ATCTCGAGGCCAGTCATCC
CDK5	Forward TCTTTTTCCCGGCAATGAT<br><br>Reverse TCTGGCAGCTTGGTCATAGA
CDK6	Forward GAACTAGGCAAAGACCTACTTCTGA<br><br>Reverse GGTGGGAATCCAGGTTTTCT
CDKN1A	Forward GTGGACCTGGAGACTCTCAG<br><br>Reverse CCTCTTGGAGAAGATCAGCCG
CDKN1B	Forward GGCTAACTCTGAGGACACGC<br><br>Reverse TGAGTAGAAGAATCGTCGGTTGC
**B-Actin***	Forward GTGGGCCGCCCTAGGCACCAG<br><br>Reverse CACTTTGATGTCACGCACGATTTC
**ENOX-2***	Forward GAGCTGGAGGGAACCTGATTT<br><br>Reverse CACTGGCACTACCAAACTGCA
**TWY-1***	Forward ATTGTCATCAAGACGCAGGGC<br><br>Reverse GTTGCGAATCCCTTCGCTGTT
**CYCR***	Forward ACTGCGGGAAGGTCTCTACTT<br><br>Reverse GGGTGCCATCGTCAAACTCTA

**UBE2D2***	Forward CCATGGCTCTGAAGAGAATCC<br><br>Reverse GATAGGGACTGTCATTTGGCC
**RNF20***	Forward GGTGTCTCTTCAACGGAGGAA<br><br>Reverse TAGTGAGGCATCATCAGTGGC
**B2M***	Forward TTGTCTTTCAGCAAGGACTGG<br><br>Reverse ATGCGGCATCTTCAAACCTCC
**GAPDH***	Forward TCAAGGCTGAGAACGGGAA<br><br>Reverse TGGGTGGCAGTGATGGCA
